# Effects of probiotics on pancreatic inflammation and intestinal integrity in mice with acute pancreatitis

**DOI:** 10.1186/s12906-023-03998-7

**Published:** 2023-05-22

**Authors:** Duangporn Werawatganon, Sarocha Vivatvakin, Kanjana Somanawat, Somying Tumwasorn, Naruemon Klaikeaw, Prasong Siriviriyakul, Maneerat Chayanupatkul

**Affiliations:** 1 Center of Excellence in Alternative and Complementary Medicine for Gastrointestinal and Liver Diseases, Department of Physiology, Faculty of Medicine, Chulalongkorn University, Bangkok, Thailand; 2 Department of Microbiology, Faculty of Medicine, Chulalongkorn University, Bangkok, Thailand; 3 Department of Pathology, Faculty of Medicine, Chulalongkorn University, Bangkok, Thailand

**Keywords:** Acute pancreatitis, Probiotics, Mice, Intestinal integrity

## Abstract

**Background:**

Severe acute pancreatitis is a potentially life-threatening disease. Despite being a common disorder, acute pancreatitis lacks a specific treatment. The present study aimed to examine the effects of probiotics on pancreatic inflammation and intestinal integrity in mice with acute pancreatitis.

**Methods:**

Male ICR mice were randomly divided into 4 groups (*n* = 6 per group). The control group received two intraperitoneal (i.p.) injections of normal saline as a vehicle control. The acute pancreatitis (AP) group received two i.p. injections of L-arginine 450 mg/100 g body weight. AP plus probiotics groups received L-arginine to induce acute pancreatitis as above. In the single-strain and mixed-strain groups, mice received 1 mL of *Lactobacillus plantarum* B7 1 × 10^8^ CFU/mL and 1 mL of *Lactobacillus rhamnosus* L34 1 × 10^8^ CFU/mL and *Lactobacillus paracasei* B13 1 × 10^8^ CFU/mL by oral gavage, respectively for 6 days starting 3 days prior to the AP induction. All mice were sacrificed 72 h after L-arginine injection. Pancreatic tissue was obtained for histological evaluation and immunohistochemical studies for myeloperoxidase, whereas ileal tissue was used for immunohistochemical studies for occludin, and claudin-1. Blood samples were collected for amylase analysis.

**Results:**

Serum amylase levels and pancreatic myeloperoxidase levels in the AP group were significantly higher than in controls and significantly decreased in probiotic groups compared with the AP group. Ileal occludin and claudin-1 levels were significantly lower in the AP group than in controls. Ileal occludin levels significantly increased, whereas ileal claudin-1 levels did not significantly change in both probiotic groups as compared with the AP group. The pancreatic histopathology showed significantly higher degree of inflammation, edema, and fat necrosis in the AP group, and these changes improved in mixed-strained probiotic groups.

**Conclusions:**

Probiotics, particularly the mixed-strain ones, attenuated AP via the reduction of inflammation and the maintenance of intestinal integrity.

**Supplementary Information:**

The online version contains supplementary material available at 10.1186/s12906-023-03998-7.

## Background

Acute pancreatitis (AP) is one of the most common gastrointestinal diseases that require hospital admission [1–3]. The most common causes of AP are gallstones and alcohol consumption [1]. Pathology of AP begins with the early conversion of pancreatic enzymes from inactive to active forms inside the acinar cells which leads to autodigestion of the pancreatic tissue. This results in the release of various proinflammatory cytokines, such as tumor necrosis factor (TNF)-α, interleukin (IL)-1, IL-6, and IL-8, and the development of pancreatic inflammation [4–6]. TNF-α also stimulates the inducible nitric oxide synthase (iNOS) in the vascular smooth muscle cells of the pancreas [7, 8]. Moreover, the excessive release of inflammatory mediators also leads to systemic inflammatory response syndrome (SIRS) and/or multiple organ dysfunction syndromes (MODS) [9]. As the clinical course of AP progresses to the late phase, local complications may appear [1]. Superimposed bacterial infection may ensue leading to the increased morbidity and mortality [2, 10].

There are many hypotheses regarding the mechanism of which infections reach the pancreas, the widely accepted one is the bacterial translocation from the intestinal tract [11–13]. Gut bacteria subsequently stimulate nucleotide-binding oligomerization domain-containing protein 1 (NOD1) and promote neutrophil priming, which in turn leads to local and systemic inflammatory responses [14]. In experimental models of AP, measures that reduced bacterial translocation prevented infectious complications of AP and improved survival [12, 15]. Nevertheless, the use of antibiotic prophylaxis in severe AP has never been shown to be effective in preventing infectious complications or reducing mortality in human studies [1, 2]. Therefore, non-antibiotic therapy should be considered. With the knowledge that bacterial translocation will occur if the protective mechanisms of the intestine which consist of both physical and immunological barriers have failed, alternative management that can strengthen the intestinal integrity might have beneficial effects in alleviating AP [16].

Probiotics are living microorganisms that when ingested in specific numbers will provide health benefits [17–19]. Examples of probiotics are bacteria, especially lactic acid bacteria and *Bifidobacteria* [18]. The lactic acid bacteria, such as *Lactobacillus plantarum* (*L. plantarum*) and *Lactobacillus rhamnosus* (*L. rhamnosus*), are believed to be effective in preserving the colonic microflora balance, eliminating toxic components, and clearing pathogens [19]. However, the use of probiotic prophylaxis in patients with AP remains controversial as some animal and human studies demonstrated potential benefits of probiotics in AP [11, 12, 20–23], whereas others have shown that probiotic prophylaxis increased the risk of bowel ischemia and mortality in patients with severe AP [24]. Therefore, the aims of this study were to evaluate the beneficial effects and mechanism of probiotic prophylaxis in a mouse model of L-arginine induced AP and to compare these effects between single- and mixed-strain probiotics. *L. plantarum* B7, *L. rhamnosus* L34, and *L. paracasei* B13 could be cultivated in our own laboratory and have been shown to have anti-inflammatory effects and reduce intestinal permeability in other conditions, such as alcoholic hepatitis [25], sepsis [26], or *Clostridioides difficile* infection [27]. As a result, we aimed to evaluate the effects of these strains of *Lactobacillus* in AP as well.

## Methods

### L-arginine preparation

L-arginine (L-arg) powder (Sigma Aldrich, St. Louis, MO, USA) was dissolved in 0.9% saline, and the pH was adjusted to 7 with NaOH. Two doses of 450 mg/100 g BW of L-arg were administered through intraperitoneal (i.p.) injections with an interval of 1 h.

### Animal protocols and specimens’ collection

Twenty-four male ICR mice, five weeks of age, and 25–30 g of weight were purchased from Nomura Siam International Co., Ltd., Bangkok, Thailand. All mice were kept under standard conditions (the room temperature at 25 ± 1ºC, room humidity 50%, and a 12-h dark–light cycle with the light on from 7:00 a.m. to 7:00 p.m.) and fed with a standard diet and water ad libitum. All mice received proper care in accordance with Ethical Principles and Guidelines for the Use of Animals by the National Research Council of Thailand, and the study was approved by Animal Care and Use Committee, Faculty of Medicine, Chulalongkorn University, Bangkok, Thailand (Approval number: 024/2562). This study was reported in accordance with ARRIVE guidelines. After one week of acclimatization, mice were randomly allocated into four groups (6 mice per group).

#### Control group

Mice were given two i.p. injections of normal saline, at an interval of 1 h as a vehicle control.

#### Acute pancreatitis (AP) group

Mice were given two i.p. injections of 450 mg/100 g BW of L-arg dissolved in normal saline, at an interval of 1 h for one day to induce AP.

#### Single-strain (S) group

Mice were given two i.p. injections of 450 mg/100 g BW of L-arg dissolved in normal saline, at an interval of 1 h. They received 1 mL of *L. plantarum* B7 1 × 10^8^ colony-forming unit (CFUs)/mL once daily via oral gavage for 6 days starting 3 days prior to the AP induction.

#### Mixed-strain (M) group

Mice were given two i.p. injections of 450 mg/100 g BW of L-arg dissolved in normal saline, at an interval of 1 h. They received 1 mL of mixed-strain probiotics (*L. rhamnosus* L34 1 × 10^8^ CFUs/mL and *L. paracasei* B13 1 × 10^8^ CFUs/mL) once daily via oral gavage for 6 days starting 3 days prior to the AP induction.

The dosages of L-arg and probiotics that we used in this study was acquired from the results of our pilot study. We did not combine *L. plantarum* with *L. rhamnosus* and *L. paracasei* as our pilot study have shown that *L. plantarum* inhibited the growth of other bacteria.

All mice were sacrificed 72 h after L-arg induction (day 3) by overdose injection of i.p. thiopental sodium. The abdomen was opened, and the whole pancreas was rapidly removed and washed with cold normal saline. Then, the ileocecal valve was identified, and 4 cm of distal ileum was removed and flushed with normal saline to remove adherent bacteria. Pancreatic and ileal tissues were fixed in 10% formalin solution immediately after collection at a room temperature for histological examination and immunohistochemical studies. Blood samples were drawn by cardiac puncture and allowed to clot by being kept at a room temperature for 2 h. Subsequently, blood samples were centrifuged at 1,000 × g for 20 min. After centrifugation, serum was collected for serum amylase analysis.

### Serum amylase analysis

The level of serum amylase was evaluated via the colorimetric method, using the biochemical analyzer Reflotron® Plus. The collected serum was applied onto a reagent strip designed specifically for quantitative amylase determination. Results were expressed as enzyme concentration in unit per liter (U/L).

### Immunohistochemistry for pancreatic myeloperoxidase (MPO) and ileal tight junction proteins

For the immunohistochemistry (IHC) of pancreatic MPO (a marker of inflammation) and ileal tight junction proteins (occludin and claudin-1), tissue microarray (TMA) technique was used. The paraffin-embedded blocks (donor blocks) were retrieved, sectioned, and stained with hematoxylin and eosin (H&E). Then, the experienced pathologist identified and marked the appropriate area on the slides. The areas on the donor block that corresponded to marked areas on the slide were cored 4–6 times. Cores were then placed in an empty paraffin block (TMA block) to make the recipient block which was used for further analyses.

The selected paraffin block was then sectioned at 5-μm thickness. The slides were deparaffinized by immersing in xylene and alcohol and then rehydrated with wash buffer. For MPO, the slides were incubated overnight with MPO goat polyclonal primary antibody at the dilution of 1:500 (R&D Systems, USA) at 4 °C. After that, they were incubated with donkey anti-goat IgG H&L (HRP) secondary antibody at the dilution of 1:500 (Abcam, UK) for 60 min at a room temperature. The slides were incubated with high sensitivity Streptavidin-HRP conjugate (HSS-HRP) for 30 min and incubated with DAB chromogen solution for 15 min. For occludin and claudin-1, the slides were incubated with occludin rabbit polyclonal primary antibody at the dilution of 1:500 (Novus Biologicals, USA) and claudin-1 rabbit polyclonal antibody at the dilution of 1:200 (Novus Biologicals, USA) at a room temperature for 1 h and incubated at 4 °C in a humidified chamber overnight. The slides were then incubated with the goat anti-rabbit IgG H&L (HRP) secondary antibody at the dilution of 1:1000 (Abcam, UK) at a room temperature for 1 h. The TMA slides were scanned using the Aperio ScanScope System (Aperio, Technology; Vista, CA) at the 40 × magnification to provide a high-resolution digital image. The analysis using the positive pixel count algorithm was performed. Positive and negative control tissues were analyzed to ensure the appropriate settings. Algorithm parameters were reported as negative, weakly positive, positive, and strongly positive. The data of pancreatic MPO, ileal occludin, and ileal claudin-1 were reported as the sum of positive and strongly positive pixels per the total number of counted pixels. The average levels of these parameters were calculated for all experimental groups [26].

### Pancreatic histopathology

After formalin fixation, the pancreatic tissue was embedded in paraffin and sectioned at 5-μm thickness. The sections were then deparaffinized with xylene and rehydrated with alcohol. Subsequently, the sections were stained with H&E. The glass slides are ready for light microscopy evaluation. An experienced pathologist evaluated all samples while being blinded to the experimental groups. Slides were evaluated for inflammation, edema, and fat necrosis and graded according to the criteria by De Cock, et al. [28]. This scoring system was selected since pancreatic inflammation and edema/fat necrosis were the main findings in acute interstitial pancreatitis of which our model represented. Inflammation grades depended on the degree of neutrophil infiltration ranging from 0–3 with 0 = No neutrophil present, 1 = mild or < 25%, 2 = moderate or 25–50%, and 3 = severe or > 50% neutrophil infiltration of the total pancreatic parenchyma. Edema and fat necrosis were also graded from 0–3 with 0 = No edema nor necrosis, 1 = mild or < 25%, 2 = moderate or 25–50%, and 3 = severe or > 50% edema or fat necrosis of the total pancreatic parenchyma.

### Statistical analysis

Statistical analysis was calculated by SPSS Statistics, Version 22.0. (Armonk, NY, USA.). All results were expressed as mean ± standard error of the mean (SEM). The difference between groups was determined by one-way analysis of variance (ANOVA) and LSD post-hoc test. Descriptive statistics were used for the histological examination of the pancreas. Differences were considered significant at *p* < 0.05.

## Results

### Serum amylase levels

**Fig. 1:**
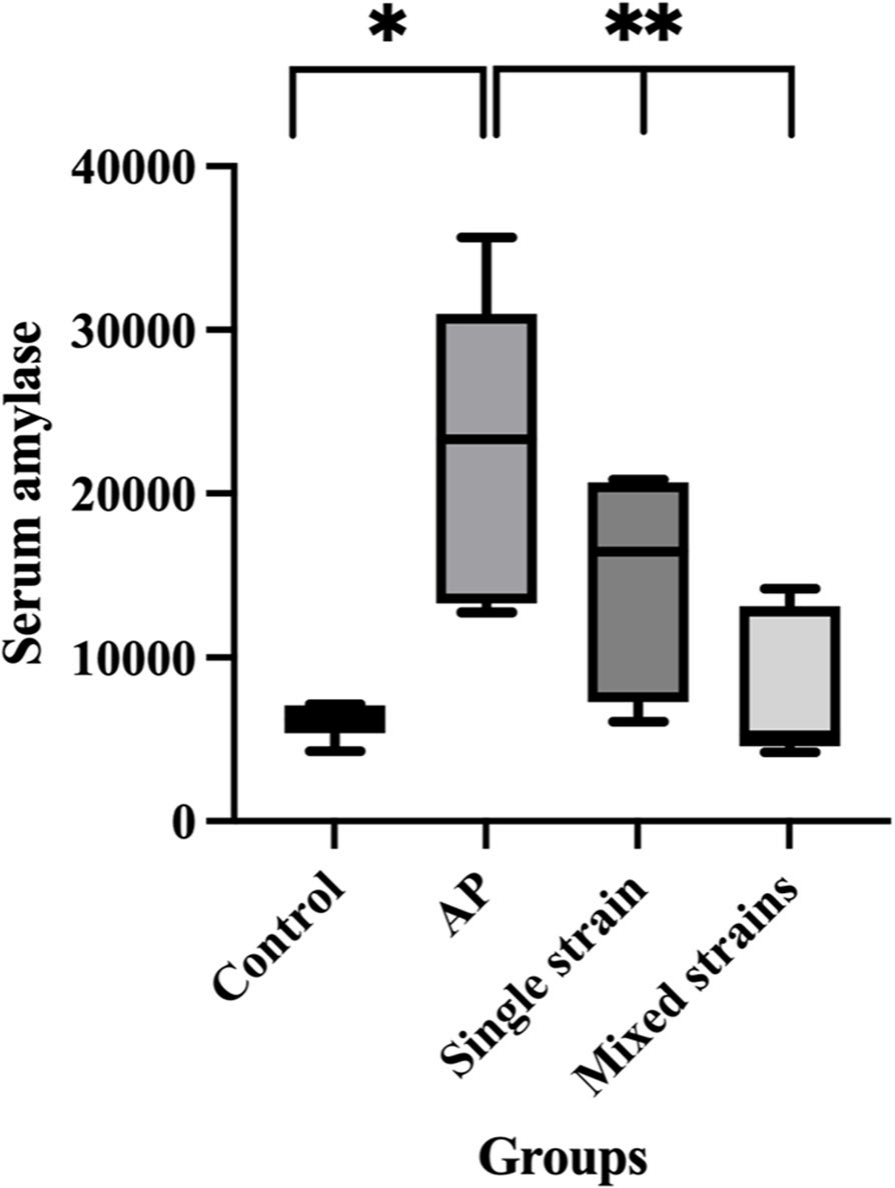
Serum amylase levels in all groups. *p < 0.05 compared with the control group; **p < 0.05 compared with the AP group

Serum amylase levels of the control and AP groups were 6,118.67 ± 428.94 U/L and 22,991.67 ± 3,720.21 U/L, respectively. Serum amylase in the AP group was significantly higher than in the control group (*p* < 0.001). Serum amylase in S and M groups were 14,681.67 ± 2,648.28 U/L and 7,730.00 ± 1,833.02 U/L, respectively. Serum amylase in both S and M groups decreased significantly compared with the AP group (*p* = 0.027 and < 0.001, respectively). Serum amylase levels were similar between S and M groups. Results of serum amylase were illustrated in Fig. 1.

### Pancreatic myeloperoxidase (MPO)

**Fig. 2:**
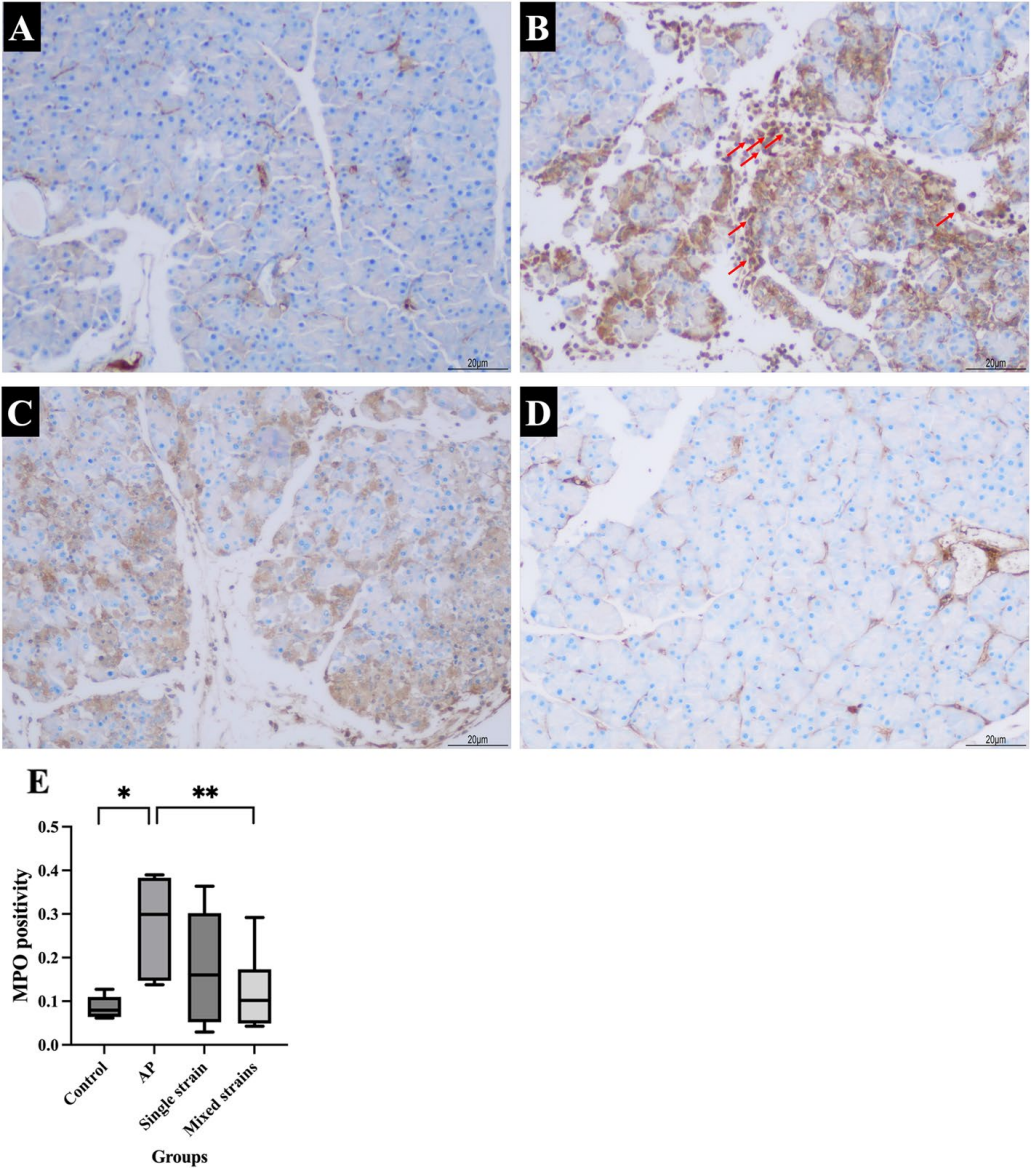
**A-E**. Representative images of immunohistochemistry for MPO (100 ×) and levels of MPO positivity in all groups. **A** IHC for the control group; **B** IHC for the AP group; **C** IHC for the S group; **D** IHC for the M group; **E** MPO levels in each group

Pancreatic MPO levels of the control and AP groups were 0.0860 ± 0.0104 and 0.2761 ± 0.0457, respectively. The MPO level in the AP group was significantly higher than in the control group (*p* = 0.004). The MPO levels in S and M groups were 0.1757 ± 0.0565 and 0.1204 ± 0.0378, respectively. The MPO level in the M group decreased significantly compared with the AP group (*p* = 0.015). MPO levels were similar between S and M groups. The results of pancreatic MPO and representative images of immunohistochemistry for MPO were illustrated in Figs. 2A-E.

Figure 2A-D showed that inflammatory cells (red arrows) increased in the AP group and decreased in mice that received probiotics. Arrows indicated inflammatory cell infiltration in the pancreatic tissue (cells with brown-stain nuclei). *p < 0.05 compared with the control group; **p < 0.05 compared with the AP group.

### Ileal occludin and claudin-1

**Fig. 3:**
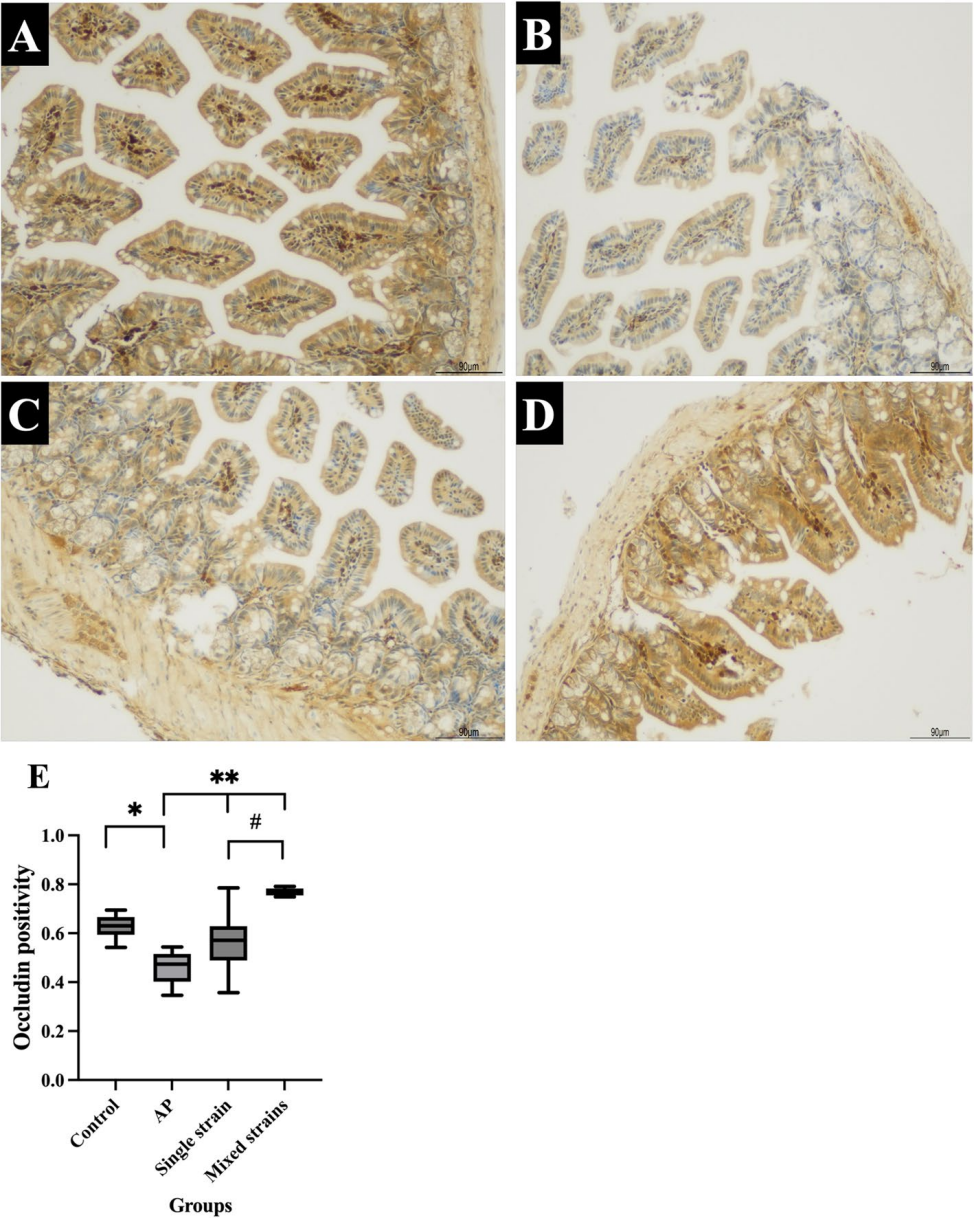
**A-E**. Representative images of immunohistochemistry for occludin (100 ×) and levels of occludin positivity in all groups. **A** IHC for the control group; **B** IHC for the AP group; **C** IHC for the S group; **D** IHC for the M group; **E** Occludin level in each group

Ileal occludin levels of the control and AP groups were 0.6277 ± 0.0210 and 0.4608 ± 0.0284, respectively. The occludin level in the AP group was significantly lower than in the control group (*p* = 0.002). The occludin levels in S and M groups were 0.5657 ± 0.0557 and 0.7693 ± 0.0067, respectively. The occludin levels in both S and M groups increased significantly compared with that in the AP group (*p* = 0.037 and < 0.001, respectively). The occludin level was significant higher in the M group than in the S group (*p* < 0.001). The results of ileal occludin and representative images of immunohistochemistry for occludin were illustrated in Fig. 3A-E.

Figure 3A-D showed that occludin positivity (brown stain) significantly decreased in the ileum of AP mice as compared with control mice and increased in mice that received probiotics. *p < 0.05 compared with the control group; **p < 0.05 compared with the AP group; ^#^*p* < 0.05 compared with the single-strain group.

**Fig. 4:**
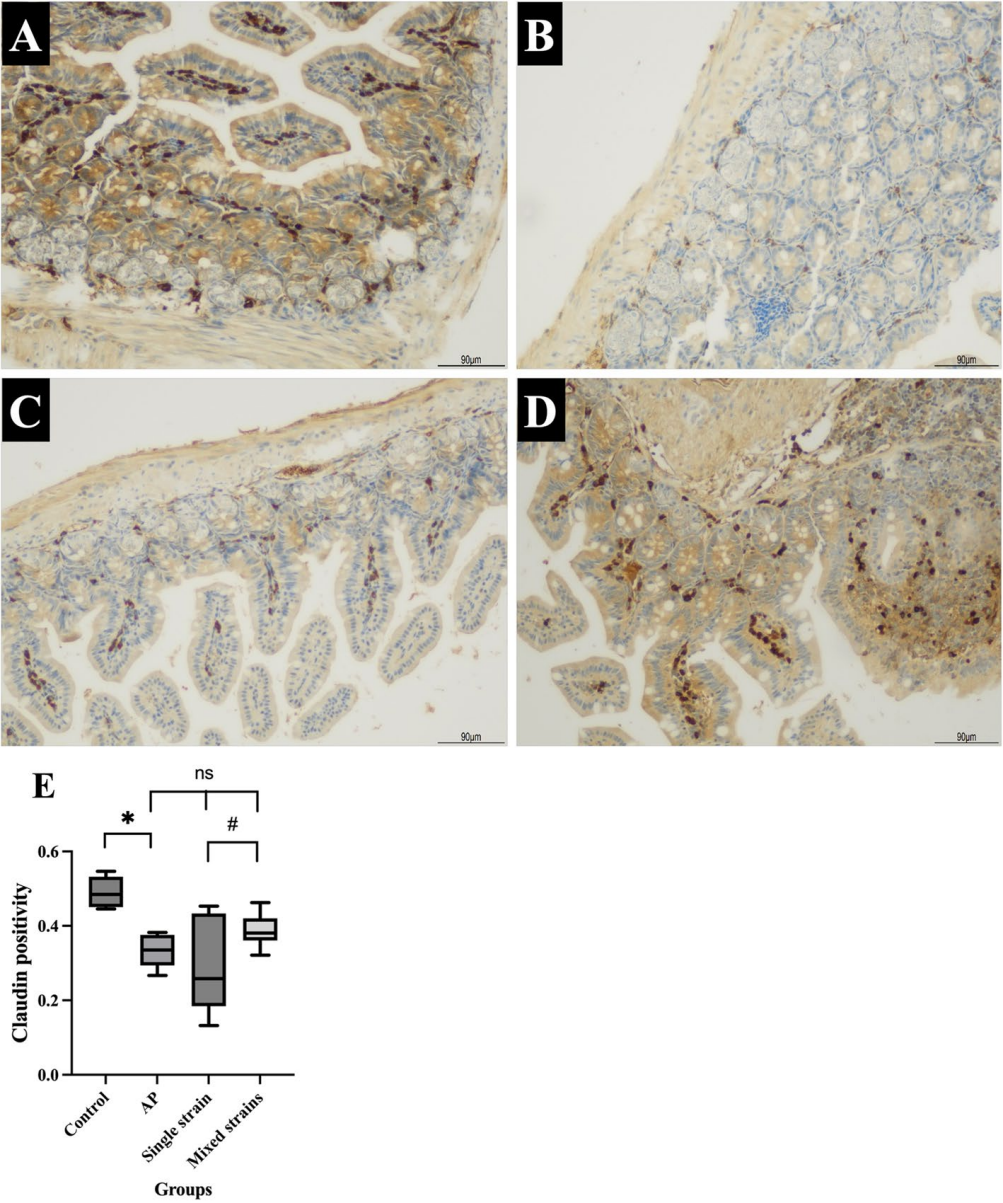
**A-E**. Representative images of immunohistochemistry for claudin-1 (100 ×) and levels of claudin-1 positivity in all groups. **A** IHC for the control group; **B** IHC for the AP group; **C** IHC for the S group; **D** IHC for the M group; **E** Claudin-1 level in each group

The ileal claudin-1 levels of the control and AP groups were 0.4904 ± 0.0178 and 0.3327 ± 0.0190, respectively. The claudin-1 level in the AP group was significantly lower than in the control group (*p* = 0.002). The claudin-1 levels in S and M groups were 0.2884 ± 0.0534 and 0.3876 ± 0.0189, respectively; these levels were not statistically different from the AP group (*p* = 0.327 and *p* = 0.227, respectively). The claudin-1 level was significantly higher in the M group than in the S group (*p* = 0.036). The results of ileal claudin-1 and representative images of immunohistochemistry for claudin-1 were illustrated in Fig. 4A-E.

Figure 4A-D showed a profound decrease in claudin-1 positivity (brown stain) in the ileum of AP mice as compared with control mice. However, there were no significant changes in the claudin-1 levels in both probiotic groups. *p < 0.05 compared with the control group; ^#^*p* < 0.05 compared with the single-strain group; ns = non-significant.

### Pancreatic histopathology

**Fig. 5:**
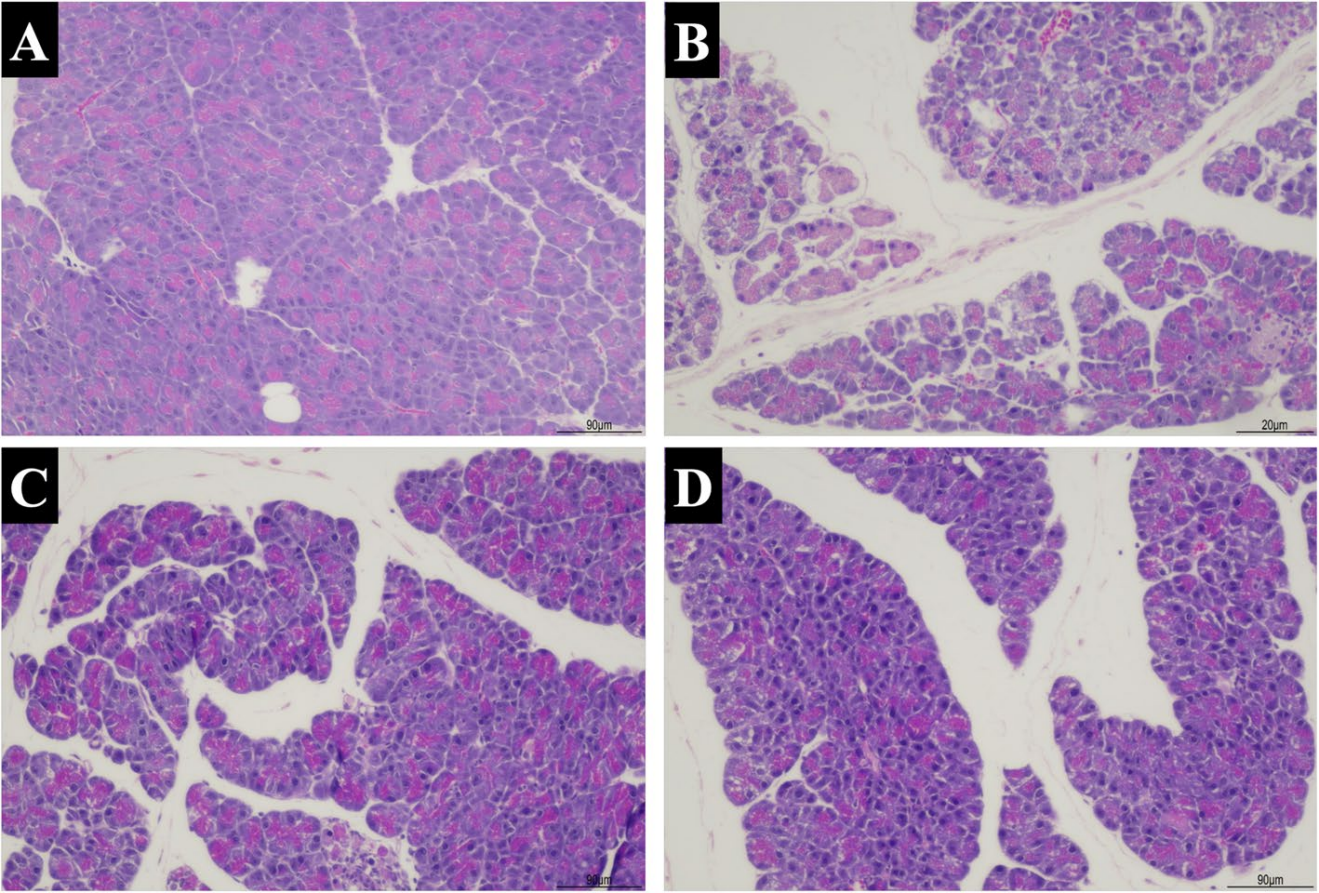
**A-D**. Representative images of pancreatic histopathology by H&E staining (100 ×) in all groups. **A** The Control group with normal histology; **B** AP group with diffuse inflammation, marked edema, and acini destruction; **C** S group with mild inflammation and edema; **D** M group with improved pathological changes

**Fig. 6:**
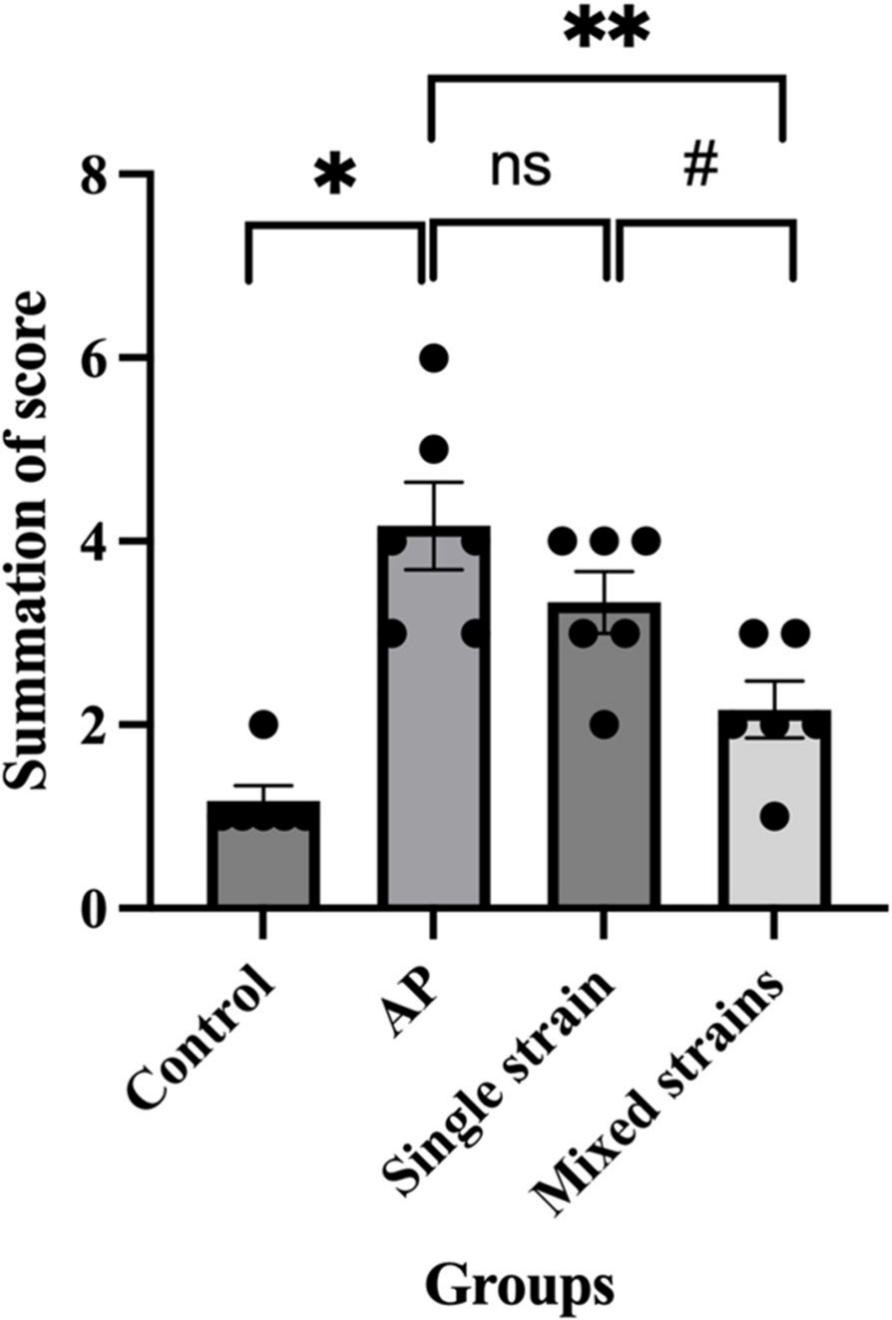
Showed the summation of pancreatic pathological scores (inflammation score + edema and fat necrosis score) in each group. Data are presented as mean ± SEM. Black dots represent the number of mice with that score in each group. *p < 0.05 compared with the control group; **p < 0.05 compared with the AP group; ^#^*p* < 0.05 compared with the single-strain group; ns = non-significant

The pancreatic histopathology of the control group revealed normal architecture with some congestions, but no inflammation was observed. In contrast, diffuse inflammation, edema, and acini destruction were found in the AP group. The AP group had higher histopathological scores compared with the control group. Both S and M groups showed the improvement of inflammation, edema and fat necrosis compared with the AP group. The M group had lower histopathologic scores than the S group. The representative images of pancreatic histopathology and histological scores of all groups were reported in Figs. 5A-D, 6, and Table 1, respectively.

**Table 1:** Summary of the pancreatic histopathological scores in each group

Group	N	Inflammation				Edema and fat necrosis			
		**0**	**1**	**2**	**3**	**0**	**1**	**2**	**3**
**Control**	6	5	1	0	0	0	6	0	0
**AP**	6	0	2	3	1	0	0	4	2
**S**	6	0	4	2	0	0	1	4	1
**M**	6	3	2	1	0	0	3	2	1

Inflammation scoring – 0 = No neutrophil present, 1 = mild or < 25%, 2 = moderate or 25–50%, and 3 = severe or > 50% neutrophil infiltration of the total pancreatic parenchyma. Edema and fat necrosis scoring – 0 = No edema nor necrosis, 1 = mild or < 25%, 2 = moderate or 25–50%, and 3 = severe or > 50% edema or fat necrosis of the total pancreatic parenchyma

The numeric represents the number of mice with that histological score

## Discussion

Acute pancreatitis (AP) can present in a wide spectrum of disease severities ranging from mild, moderate, and severe AP [29]. Severe AP is defined according to the modified Marshall scoring as AP with persistent organ failure of more than 48 h and usually in conjunction with local complications, such as infected pancreatic necrosis which is resulted from bacterial translocation. Severe AP is strongly associated with high morbidity and mortality rates [2, 10]. Therefore, the prevention of bacterial translocation could potentially attenuate the severity of AP and thus, reducing the mortality rate. Clinical studies have shown that probiotics could reduce the length of hospital stay in patients with AP; however, their effects on mortality and infectious complications remain conflicting [30, 31].

The diagnosis of AP in this study was made based on pancreatic histopathological changes and the elevation of serum amylase. Amylase is an enzyme produced by the acinar cells of the pancreas. In AP, acinar cell injury occurs, and thus, serum amylase is released from the injured acinar cells. Serum amylase rises rapidly 3–6 h after the onset of symptoms and lasts for 3–5 days [2]. In this study, serum amylase was significantly risen in the AP group compared to the control group as expected. Furthermore, mice that received probiotic pre-treatment whether as single- or mixed-strain probiotics had significantly lower serum amylase levels compared with mice in the AP group with a more prominent effect in the mixed-strain probiotic group. Similar to our results, Aykol and colleagues showed the decline of serum amylase levels in rats that received *Saccharomyces boulardii* and antibiotics compared with antibiotics alone [32]. Li and colleagues similarly showed that the colonization by *B. animalis* in germ-free and antibiotics-treated mice protected these mice again AP as evidenced by the reduction in serum amylase and pancreatic pathological changes [33]. In contrast, other studies demonstrated that rats receiving Ecologic 641, multiple-strain probiotics that consist of *L. acidophilus*, *L. casei*, *L. salivarius*, *L. lactis*, *B. bifidum*, and *B. lactis*, showed no differences in serum amylase levels compared with control rats [12, 20]. Another study using a different mixture of probiotics containing *L. casei*, *L. rhamnosus*, *L. acidophilus*, and *B. longum* also showed no differences in serum amylase levels between rats that received and did not receive probiotics [34]. We hypothesized that probiotic administration attenuated the severity of acinar cell damage, and thus, reducing serum amylase levels [32]. However, the inconsistency of findings between studies might be because of the differences in strains and strength of probiotics used.

Mice in the AP group showed significant changes in pancreatic histopathology as compared with those in the control group. Mice receiving mixed-strain probiotics showed an improvement in histological scores; in contrast, mice receiving single-strain probiotics did not show significant improvement. Our results were in contrast with findings from a study by van Minnen and colleagues. The authors found that probiotic supplement, despite reducing late mortality and improving clinical course, did not lead to significant histological improvement compared with placebo. In that study, they used multi-strain probiotics at the concentration of 5 × 10^9^ CFU/mL starting 5 days prior to AP induction compared to our study that used probiotics concentration at 1 × 10^8^ CFU/mL starting 3 days prior to AP induction [11]. Additionally, a meta-analysis of 6 studies on experimental AP demonstrated that probiotic supplement reduced total histopathological scores compared with controls [35]. The differences in histological outcomes could be potentially explained by the different duration of probiotic supplement (from 3 to 14 days prior to AP induction), the species of animals (mice vs. rats), the timing of histological assessment (from 12 h to 4 days after AP induction), probiotic strains and strength (2.4–5.0 × 10^9^ CFU/mL). A previous study suggested that a longer duration of probiotic pre-treatment before AP induction might be needed to see positive results on pancreatic histopathology because a short duration of probiotic supplement might not have a significant impact on gut microbiome [36]. However, higher dose of probiotics and longer duration of therapy did not always translate into better outcomes as evidenced by a study by Horst and colleagues which showed that giving probiotic mixture 1.2 × 10^9^ CFU/mL 14 days prior to the induction of AP led to greater pancreatic tissue damage than in the AP-alone group [34].

Neutrophil recruitment and activation plays a crucial role in the development of pancreatic inflammation and injury in the early phase of AP [37]. As MPO is mainly expressed in neutrophils and is important to neutrophil functions [38], pancreatic tissue MPO was used as a marker of neutrophil infiltration and inflammation in this study. We found that pancreatic MPO levels were significantly higher in the AP group than in the control group and improved with mixed-strain probiotic supplement. These results indicated that probiotic supplement could alleviate pancreatic inflammation, which was in accordance with the reduction in serum amylase and the degree of inflammation on pancreatic histology in the mixed-strain group. Similar to our results, Takauji and colleagues demonstrated that *Lactobacillus brevis* SBL88-derived long chain polyphosphate significantly reduced the numbers of MPO-positive cells and inflammatory cytokine expression in pancreatic tissues of mice with cerulean-induced AP [36].

Small bowel bacterial overgrowth, mucosal barrier failure, and impaired host defense mechanism are the three main steps involved in the development of bacterial translocation and infectious complications in AP [11, 12]. Early after the onset of AP, neurohormonal substances that are released in response to AP reduce small bowel motility leading to the stasis of luminal contents and overgrowth of potential enteric bacteria including *E. coli* and *Enterococcus spp.* [39]. These luminal pathogens along with AP-induced reduction of intestinal blood flow result in mucosal ischemia and reactive oxygen species (ROS) release [21]. Subsequent development of oxidative stress leads to the release of pro-inflammatory mediators and the disruption of epithelial tight junctions resulting in increased epithelial permeability, and eventually pathogenic bacterial translocation [40]. In accordance with previous reports, our findings showed that ileal occludin and claudin-1 levels significantly decreased in mice with L-arg-induced AP indicating the disruption of intestinal integrity. Probiotic supplement with either single or mixed strains of *Lactobacillus* increased ileal occludin, but not claudin-1 levels compared with the AP group. It is important to note that ileal occludin and claudin-1 levels were significantly higher in the mixed-strain group than in the single-strain one. Similar to our results, Takauji and colleagues found that pre-treatment with *Lactobacillus brevis*-derived polyphosphate significantly increased colonic occludin expression in cerulein-induced AP mice leading to the reduction in the intestinal permeability. Colonic claudin-1 expression was also augmented in the treatment group albeit not statistically significant [36]. It is hypothesized that probiotics strengthen intestinal barrier through the stimulation of glutathione production in the intestine, which in turn reduces oxidative stress induced mucosal damage [21].

Our study was not without limitations. Firstly, we did not perform gut microbial analysis in each group so we could not state with certainty that probiotic supplement protected against AP through the modulation of gut microbiota. Further studies are needed to confirm this mechanism. Secondly, probiotics were given to mice 3 days prior to the AP induction until 3 days afterward. Pre-treatment protocol might not be completely applicable to the clinical setting as patients usually present to the hospital after AP has already developed. Nevertheless, this protocol could still be applied to patients with recurrent AP from genetic diseases to see if probiotic supplement could prevent pancreatitis episode. Clinical studies are warranted to prove its benefits in this aspect.

## Conclusions

Probiotic supplement, particularly the mixed-strain ones, alleviated the severity of L-arg-induced AP through the reduction of inflammatory responses and the improvement of intestinal integrity.

## Supplementary Information

**Additional file 1: Supplementary 1.** - Sample size calculation.

## Data Availability

All data generated or analysed during this study are included in this published article and its supplementary information file.

## Abbreviations

AP: Acute pancreatitis
BW: Body weight
CFU: Colony-forming unit
H&E: Hematoxylin and eosin
IHC: Immunohistochemistry
IL: Interleukin
iNOS: Inducible nitric oxide synthase
i.p: Intraperitoneal
L-arg: L-arginine
*L. **plantarum*: *Lactobacillus* *plantarum*
M group: Mixed-strain group
MODS: Multiple organ dysfunction syndromes
MPO: Myeloperoxidase
NOD1: Nucleotide-binding oligomerization domain-containing protein 1
S group: Single-strain group
SIRS: Systemic inflammatory response syndrome
TMA: Tissue microarray
TNF-α: Tumor necrosis factor-α
