# Supplementary material for: Effects of probiotics on pancreatic inflammation and intestinal integrity in mice with acute pancreatitis
Source: BMC Complement Med Ther. 2023 May 22;23:166. doi: 10.1186/s12906-023-03998-7 (PMC10204250; doi:10.1186/s12906-023-03998-7)
Supplement: Supplementary file 1 — Additional file 1: Supplementary 1. - Sample size calculation. [file 12906_2023_3998_MOESM1_ESM.docx]

**Supplementary 1 - Sample size calculation**

The number of mice per group was calculated using the data from the study by Sriko et al. The authors demonstrated that genistein, a chemical compounds derived from plants was effective in the treatment of L-arginine-induced acute pancreatitis in mice. The concentration of serum amylase determined by the biochemical analyzer Reflotron® Plus (mean ± SD) in each group was as follows; group 1: normal (n=6) 5,714.00 ± 201.11 U/L; group 2: L-arginine induced acute pancreatitis (n=6) 13,860.00 ± 5,918.26 U/L; group 3: L-arginine induced acute pancreatitis + genistein (n=6) 8,728.33 ± 3,213.61 U/L. From these results, we used mean and SD in each group to calculate sample size by using G*Power 3.1.9.2 with the alpha of 0.05 and power of 0.98 (image shown below). The analysis revealed the total sample size of 24 rats rendering the number of rats per group of 6.


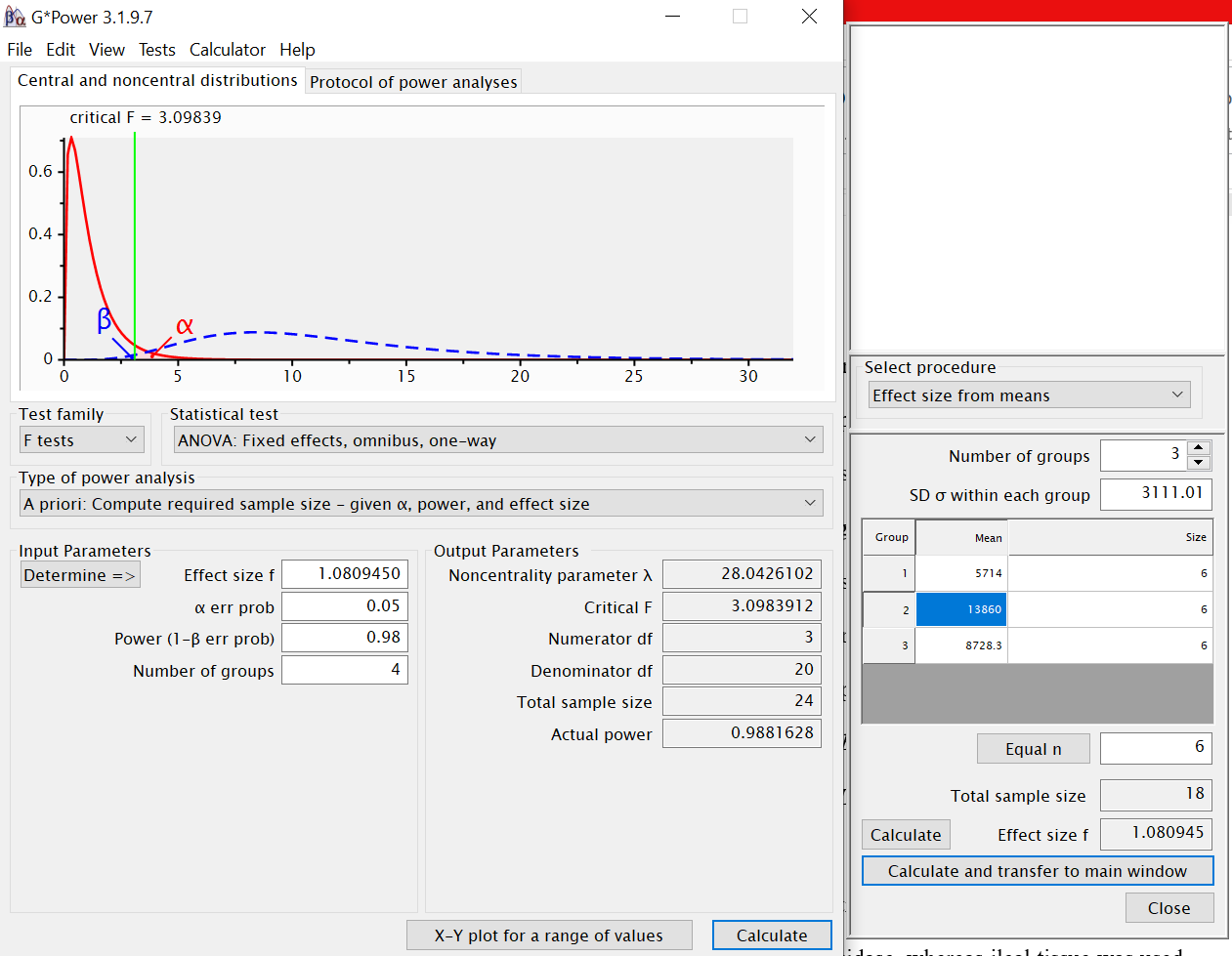


References: Sriko J, Werawatganon D, Klaikaew N, Siriviriyakul P. Genistein Attenuated Severity of Acute Pancreatitis Induced by L-Arginine in Mice. *J Physiol Biomed Sci*. 2018; 31(1):12-17.

Dataset is available at <https://docs.google.com/spreadsheets/d/1wBgr8NqGScrKIpBzlWI_3ujFfsJG6_QF/edit?usp=share_link&ouid=110678368276930470974&rtpof=true&sd=true>
